# Au and AuCu Nanoparticles Supported on SBA-15 Ordered Mesoporous Titania-Silica as Catalysts for Methylene Blue Photodegradation

**DOI:** 10.3390/ma11060890

**Published:** 2018-05-25

**Authors:** Isabel Barroso-Martín, Elisa Moretti, Aldo Talon, Loretta Storaro, Enrique Rodríguez-Castellón, Antonia Infantes-Molina

**Affiliations:** 1 Departamento de Química Inorgánica, Cristalografía y Mineralogía (Unidad Asociada al ICP-CSIC), Facultad de Ciencias, Universidad de Málaga, Campus de Teatinos, 29071 Málaga, Spain; isabel.barroso@uma.es (I.B.-M.); castellon@uma.es (E.R.-C.); 2 Dipartimento di Scienze Molecolari e Nanosistemi, Università Ca' Foscari Venezia, National Interuniversity Consortium of Materials Science and Technology (INSTM) Venice Research Unit, Via Torino 155/B, 30172 Mestre Venezia, Italy; aldair@unive.it (A.T.); storaro@unive.it (L.S.)

**Keywords:** pollutant degradation, methylene blue, photocatalysis, SBA-15 mesoporous silica, nanoparticles (NPs), Au NPs, AuCu NPs

## Abstract

The photocatalytic degradation of methylene blue (MB) dye has been performed under UV irradiation in aqueous suspension, employing photocatalysts based on Au (1.5 wt %) and AuCu (Au/Cu = 1, 2.0 wt %), and supported on SBA-15-ordered mesoporous silica, with and without titania (Si/Ti = 3), in order to evaluate the versatility of this mesoporous support in this type of reaction of great impact from the environmental point of view. Samples were characterized by X-ray diffraction (XRD), transmission electron microscopy (TEM), N_2_ adsorption-desorption at −196 °C, and X-ray photoelectron spectroscopy (XPS), so as to study their structural, optical, and chemical properties. All the prepared catalysts were found to be active in the test reaction. The bimetallic AuCu-based catalysts attained very high MB degradation values, in particular AuCu/SBA-15 titania-silica sample reached 100% of dye oxidation after the monitored reaction period (120 min).

## 1. Introduction

Photocatalysis plays a significant role in air, water, and pollutant degradation. Due to the heavy industrialization the modern world is experiencing nowadays, water remediation, involving water cleaning and sanitation, is a serious environmental issue for both human and aquatic life, as a notorious number of industries releases industrial effluents during their regular activity, that constitute a threat to water quality. These effluents contain refractory, toxic, and potentially carcinogenic organic and inorganic compounds, employed either as raw materials or as reaction intermediates, that are chemically stable and non-biodegradable. Pollutants, such as heavy metals, pesticides, solvents, or dyes, the latter responsible for a major visual impact due to their bright and long-lasting colors, not only obstruct light penetration (and hence reduce photosynthetic activity of aquatic life), but also disturb the food chain of the water ecosystem [1], and cause clean water scarceness [2]. About 15% of the total world production of dyes is lost during the dyeing process and is discharged as textile effluents. The release of those colored dyes in wastewaters is disrupting the ecosystem. Originally, dye photodegradation was extensively studied by a real need to treat contaminated water effluents from textile, leather tanning, and paper industries, among others [3,4,5]. Nonetheless, since the start of self-cleaning surfaces [6,7], dyes rose as means for demonstrating photocatalysis potential. International Organization for Standardization (ISO) 10678:2010 [8] specifies a method for the determination of the surface photocatalytic activity by methylene blue (MB) degradation in aqueous solution with artificial ultraviolet (UV) radiation. The MB test is so-claimed to be appropriate to assess the ability of a photocatalyst to purify water. The test method is also applicable to the evaluation of the specific photocatalytic self-cleaning activity of surfaces covered with respective coatings. As a matter of fact, the Japanese industrial standard JIS-R-1703-2:2007 employs methylene blue for evaluating self-cleaning surfaces [9].

Although titania is, by far, one of the best photocatalysts, not only for its outstanding photocatalytic activity, but also because it is a widely spread, economical material, greatly thermal and chemically stable, and with no toxicity [10,11,12], its wide band gap (3.2 eV for the most active phase, anatase [13]) has limited its use to the UV region, which accounts for only ca. 5% of the total incident solar spectrum [14]. Doping titania with both metal and non-metal elements is one of the most used strategies to modify its band-gap, obtaining the best results when using noble metals, thanks to their exceptional optical properties [15,16], especially when using noble metal nanoparticles (NPs) [17,18], as these favor charge separation, as well as extending light absorption. The synergy between noble metals and semiconductor photocatalysts can involve many significant changes to photocatalysis. An enhancement in reactivity was, for the first time, observed for water splitting on Pt/TiO_2_ systems [19,20]*.* With a view to develop industrially scalable processes, Au NPs are also presented as a promising alternative to obtain highly active photocatalysts when operating at mild conditions (atmospheric pressure and temperature between 30 and 200 °C) [21,22]. Furthermore, gold can form alloys with different metals, improving its photocatalytic properties and showing, in oxidation processes, a mechanism similar to monometallic NPs deposited on TiO_2_, but enhancing the synergistic effects [23]*.* On the other hand, photoactivity is highly dependent on several parameters, most of them correlated, such as crystallinity, specific surface area, or pore size distribution of the TiO_2_ [18,24]. As titania specific surface area is relatively low, various synthesis methods have been developed in order to control the surface area, as well as the surface exposed and pore size distribution, where the most relevant methods are chemical vapor deposition [25], sol-gel method [26], and hydrothermal or solvothermal methods [27,28], among others [29]. Likewise, specific surface area can be improved by using surfactants like cetyltrimethylammonium bromide (CTAB) [30], achieving surface areas more than twice larger than that of commercial P25 titania. Nonetheless, titania specific surface area can be substantially increased by incorporating TiO_2_ active species in the internal surface of a mesoporous structure with a considerably high specific surface area like SBA-15 silica, obtaining titania-silica nanocomposites with high surface area values [31]. This is due to the fact that SBA-15 ordered mesoporous silica has unique properties, allowing its successful use in many different applications, including drug delivery, biomedical, diagnostic, engineering catalyst supports, separation of proteins, enzyme immobilization [32,33,34] and, thanks to its excellent adsorption ability, adsorption of heavy metal ions [35], organic dyes [24], polycyclic aromatic hydrocarbons [36], and other organic pollutants.

In this context, this paper aims at the study of the photocatalytic performance in the degradation on methylene blue at ambient conditions under UV irradiation of Au photocatalysts supported on SBA-15 mesoporous silica and titania-silica. In addition, it has been investigated the influence of adding a second metal, such as copper, in order to investigate possible synergistic effects of AuCu bimetallic system in the studied reaction.

## 2. Materials and Methods

### 2.1. Materials

The chemical products employed for the synthesis of both supports and catalysts are commercially available and were used without further purification. H_2_SO_4_ 95%, VWR (Radnor, PA, USA); NaOH 97%, VWR; Pluronic (poly(ethylene glycol)-block-poly(propylene glycol)-block-poly(ethylene glycol)), Aldrich (St. Louis, MO, USA); sodium silicate solution, reagent grade, Sigma-Aldrich (St. Louis, MO, USA); APTES ((3-aminopropyl)triethoxysilane) > 98%, Sigma-Aldrich; C_2_H_5_OH 96% vol, VWR; CTAB (Hexadecyltrimethylammonium bromide) > 98%, Sigma (St. Louis, MO, USA); TBOT (titanium (IV) butoxide) > 97%, Aldrich; NaBH_4_ 98%, Sigma-Aldrich; Cu(NO_3_)_2_·3H_2_O 99.5%, Merck; HAuCl_4_·3H_2_O 99.9%, Sigma-Aldrich.

### 2.2. Synthesis of Catalysts

SBA-15 mesoporous silica was synthesized using the method described by Cazalilla et al. [37]. The incorporation of Ti to the SBA-15 was accomplished following the post-synthesis method reported by Shindo et al. [38], employing a nominal Si/Ti molar ratio equal to 3. These supports were functionalized with aminopropyltriethoxysilane, APTES, (H_2_N(CH_2_)_3_Si(OEt)_3_), according to the procedure described by Tu et al. [39] in order to improve both the incorporation and dispersion of Au nanoparticles. Thus, in a typical synthesis, 2.5 g of APTES, 50.0 g of ethanol and 1.0 g of support were mixed and refluxed for 24 h. After filtration, washing, and drying, the APTES-grafted SBA materials were obtained. Au samples where synthesized following the procedure described by Liu et al. [40]: the supports were dispersed in water before the addition of a 10 mM solution of tetrachloroauric acid (HAuCl_4_). The suspension was maintained in stirring for two hours and then it was filtered and washed with water before being redispersed in water, reduced with NaBH_4_, and recovered by filtration. Finally, the samples were dried at 60 °C overnight, and calcined at 500 °C for 6 h. The samples were referred to as Au/Si and Au/TiSi. AuCu supported on Ti-SBA and SBA materials were prepared in a similar way. Thus, once the solid above-described was recovered by filtration after NaBH_4_ reduction, a certain amount of Cu(NO_3_)_2_ solution was added to water-redispersed APTES-functionalized Au samples and kept stirring for two hours followed by the solid recovery and a second reduction with NaBH_4_. Analogously, the samples were dried at 60 °C and calcined at 500 °C for 6 h. A reduction with H_2_ at 500 °C for 2 h was required to obtain the AuCu alloy. The volume of HAuCl_4_ and Cu(NO_3_)_2_ solutions was calculated to obtain Au catalyst with a metal loading of 1.5 wt %. In the case of AuCu samples, the total Au + Cu nominal loading was 2.0 wt % with Au/Cu molar ratio equal to 1. Samples were referred to as AuCu/Si and AuCu/TiSi.

### 2.3. Characterization of Catalysts

X-ray powder diffraction (XRPD) patterns were collected on a PAN analytical X’Pert Pro automated diffractometer. Powder patterns were recorded between 0.5° and 70° in 2θ, with a step size of 0.0167° (2θ) and an equivalent counting time of ~60 s/step, in Bragg-Brentano reflection configuration by using a Ge (111) primary monochromator (Cu K α1) and the X’Celerator detector.

N_2_ physisorption measurements were performed at −196 °C with an ASAP 2010 apparatus of Micromeritics. After outgassing at 130 °C for 12 h at 0.67 Pa, the N_2_ isotherms were acquired to determine the specific surface areas through the BET equation (S_BET_), the specific pore volume (Vs) calculated at P/P_0_ = 0.98, and pore size distribution by DFT method.

The diffusive reflectance UV-vis (DRUV-vis) spectra were collected with a Perkin Lambda 35 UV-vis spectrophotometer (PerkinElmer, Waltham, MA, USA), equipped with integrating sphere accessory, with the wavelength ranging from 300 to 800 nm. The absorption coefficient (α) was calculated as follows: α = ln(1/T)/d, where T is the measured transmittance and d is the optical path length. Band gap energy, Eg, was determined thorough the α value (m^−1^) from a plot of (αhυ)^1/2^ versus photon energy (hυ), where h is Planck’s constant and υ is the frequency (s^−1^). The intercept of the tangent to the absorption curves was used to estimate the band gap (Eg) value.

High resolution transmission electron microscopy (HR-TEM) was performed by using a TALOS F200x instrument (Thermo Fisher Scientific, Waltham, MA, USA). TEM analysis was performed at 200 kV and 5.5 µA and scanning transmission electron microscopy (STEM) with a HAADF detector was carried out at 200 kV and 200 pA. ImageJ software (ImageJ 1.48v) was used to estimate the average particle size distribution.

X-ray Photoelectron spectra (XPS) were collected using a Physical Electronics PHI 5700 spectrometer (Physical Electronics, Inc., Chanhassen, MN, USA) with non-monochromatic Mg Kα radiation (300 W, 15 kV, 1253.6 eV) for the analysis of the core level signals of C 1*s*, O 1*s*, Si 2*p*, Ti 2*p*, and Au 4*f*, and with a multichannel detector. Binding energy (BE) values were referenced to the C 1*s* peak (284.8 eV) from the adventitious contamination layer. The spectrometer energy scale was calibrated using Cu 2*p*_3/2_, Ag 3*d*_5/2_, and Au 4*f*_7/2_ photoelectron lines at 932.7, 368.3, and 84.0 eV, respectively. The PHI ACCESS ESCA-V6.0 F software package and Multipak v8.2b were used for acquisition and data analysis, respectively. A Shirley-type background was subtracted from the signals. Recorded spectra were always fitted using Gauss-Lorentz curves, in order to determine the binding energy of the different element core levels more accurately. The error in BE was estimated to be ca. 0.1 eV.

### 2.4. Photocatalytic Activity

The degradation of methylene blue, MB, was chosen as a test reaction to evaluate the photocatalytic activity of the synthesized materials under UV irradiation [8]. A 100 mL Pyrex photochemical reactor with a 125 W high pressure mercury lamp (model UV13F, Helios Italquartz, Cambiago, Italy), operating at wavelengths between 180 and 420 nm with a peak at 366 nm, was used. The initial concentration of the target molecule was 6.0 × 10^−5^ M. The amount of the photocatalyst was fixed at 1.25 g·L^−1^. All the degradation experiments were carried out at 20 °C. The photon flux was measured by using a DeltaOHM (Selvazzano Dentro, Italy) radiometer HD2302.0 leaned against the external wall of the photoreactor containing only pure water. To reach the adsorption equilibrium before irradiation, the suspension was stirred in the dark for 30 min. After switching on the lamp, aliquots of 2 mL of the aqueous suspension were collected from the reactor and filtered through a 0.45 μm PTFE Millipore disc to remove the catalyst powder.

A Shimadzu UV-2450 UV/Vis spectrometer (Mason Technology, Dublin, Ireland) was used for the determination of the dye concentration, after calibration. The degradation processes were monitored following the absorbance at the maximum (660 nm) of the UV-vis spectrum of the target molecule. Since the degradation pathway for the selected dye is known with high reliability [24,41], the eventual formation of byproducts was checked, monitoring the overall UV-vis spectrum of the solutions recovered at different times during the degradation experiments.

The rate constant *k* was calculated according to the following Equation (1):

(1) $$ln\frac{C}{C_{0}}=-kt$$

where *k* is pseudo first order rate constant (min^−1^), *C* is concentration after time *t*, and *C*_0_ represents the initial concentration and is calculated as (2):

*k* = 2.303 × slope (2)

## 3. Results

### 3.1. Photocatalytic Activity

Methylene blue, a heterocyclic aromatic organic compound, is one of the most well-known organic dyes that largely contribute to the dye pollution in water source. Therefore, it was taken as the photocatalytic probe molecule in this work. It is well known that the pH value influences the rate of photocatalytic degradation of some organic compounds [42]. In the case of a charged dye, the quantities adsorbed in the dark depend on both the surface area and the point of zero charge (PZC) or isoelectric point (IEP) of the materials. SBA-15 silica has the PZC at about pH 4 [43], while that of titania is around pH 6.8 [44], which gives rise to a negative charge for pH values higher than the PZC on the surface of the oxide particles, with an electrostatic absorption between the negatively charged surfaces of SiO_2_ or TiO_2_ and the cationic dyes. As the pH of the system decreases, the number of surfaces with positive charge increases, and the surface sites on the oxide do not tend to absorb the cations of dyes due to electrostatic repulsion.

In the case of the TiO_2_-SiO_2_ composites, PZC values usually higher than 4 pH units were reported [45]. Therefore, in our operating conditions (slightly acidic pH), the surface of the catalysts is deprotonated and covered by negatively charged moieties, more so on the pure silica catalysts than on the titania-silica ones, so that the cationic MB dye can be preferentially adsorbed on the SiO_2_-exposed surface. The pathway for MB degradation under UV irradiation on TiO_2_-containing materials is well known and has been studied in detail by means of LC/MS and GC/MS analyses of the intermediate compounds [41].

**Figure 1:**
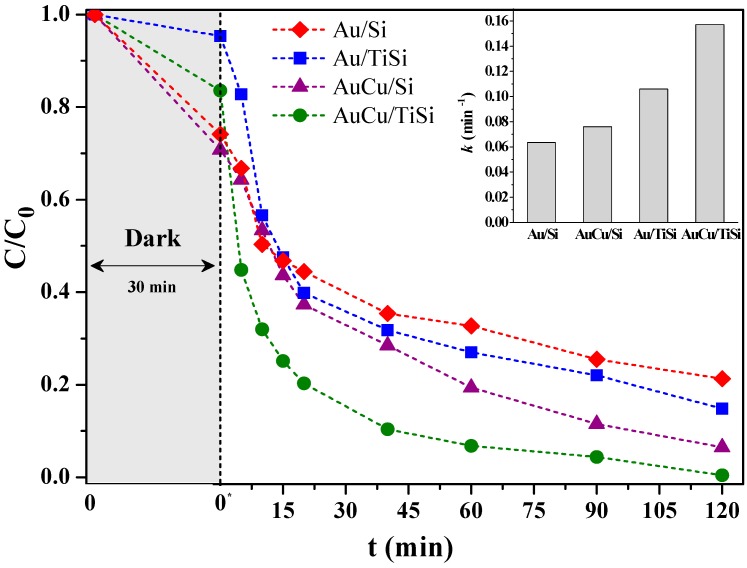
Performances of adsorption and methylene blue (MB) photodegradation on mesoporous silica and titania-silica-based catalysts before and under UV irradiation, with the calculated kinetic constants (inset).

Figure 1 shows the kinetics of disappearance of MB concentration as a function of time during photocatalytic experiments under UV irradiation for the investigated materials. Pseudo-first order kinetic constants were calculated, plotting ln(*C*_0_/*C*) as a function of time for the first part of the exponential decay curves. The values of the kinetic constants *k* are reported in the inset of Figure 1.

After 30 min of equilibration in the dark, a certain amount of MB is adsorbed on the surface of the materials, with an adsorption of about 30% for the silica-based catalysts and of a lower amount for the titania-silica-based samples. This behavior can be related with the above described selective adsorption of MB.

Under UV light irradiation, SBA matrix does not present any photocatalytic activity, and the MB catalytic removal on Ti-SBA support is rather low, with a degradation kinetic constant *k* of 0.02 min^−1^. The incorporation of metal nanoparticles onto these supports enhances the photocatalytic activity and, after switching on the UV lamp, the concentration of MB decreases exponentially with time for all the NP-containing materials. As expected, loading the two mesoporous silica-based supports, Si and TiSi samples, with 1.5 wt % of Au nanoparticles, shows a positive effect on the performance of the photocatalysts. After 120 min of UV light irradiation, Au/Si and Au/TiSi samples are able to oxidize the dye of about 79% and 85%, respectively.

Under UV-visible illumination, two different phenomena have to be taken into account at the interface metal NPs/metal oxide during a photochemical process: Schottky barrier formation and/or surface plasmonic resonance (SPR). If a metal NP/semiconductor junction is established, besides the SPR phenomenon, the difference in their Fermi levels introduces a Schottky barrier between the metal and the semiconductor, diminishing the rate of recombination between electrons and holes, and favoring, in this way, the photocatalytic activity [46]. In our case, under UV light, Au NPs act as an electron relay, able to improve the MB photo-oxidation, and when they are in contact with TiO_2_, which is a semiconductor, a Schottky barrier is formed between them, playing a more significant role in enhancing the photoactivity of the reaction compared to the SPR phenomenon [47].

In fact, in the presence of a Schottky barrier, electrons are effectively trapped into the metal, unable to flow back to the titania. In this way, the metal acts a sort of electron sink for the photoinduced electrons, preventing the e^−^/h^+^ pairs recombination, and prolonging their life.

It is evident that the presence of TiO_2_ into the silica network plays a significant role in the increase of the degradation rate constant *k*, which follows the trend AuCu/TiSi > Au/TiSi > AuCu/Si > Au/Si (see inset of Figure 1), with *k* values of 0.16, 0.11, 0.08, and 0.06 min^−1^, respectively. The simultaneous presence of both TiO_2_ and metal NPs finely dispersed on a high porous SBA-15 silica is needed to achieve a NP/semiconductor junction and enhance the *k* of the reaction. Therefore, it can be noticed that both monometallic Au and bimetallic AuCu, supported on pure silica SBA-15, photocatalyze, to a lesser extent, the MB degradation under UV irradiation. Gold nanoparticles, in fact, strongly absorb ultraviolet light, causing the transition of 5*d* electrons to the 6*sp* band, allowing the oxidation reaction of a dye [48].

Regarding the amount of MB oxidized in a relatively long period (120 min), the trend seems, in some extent, different: AuCu/TiSi > AuCu/Si > Au/TiSi > Au/Si.

Regarding the AuCu-based catalysts, the first important observation is that the two photocatalysts attain very high MB degradation values within the monitored reaction period. In particular, AuCu/TiSi reaches a 93% of dye oxidation just after 60 min and 100% after 120 min, as shown in the inset of Figure 1. A higher photocatalytic dye conversion in bimetallic AuCu samples during the monitored period is due to the well-known decrease of the work function of the bimetallic systems with respect to the monometallic components, leading to the prevention in the recombination rate of charge carriers [46].

The sample showing the highest photocatalytic performance, AuCu/TiSi, was chosen for a reusability test. After the first catalytic cycle, MB was introduced in the reactor restoring the initial concentration. The degradation of the dye was monitored during two consecutive cycles (not shown), observing a slight decrease in the photocatalytic performances of the material, most probably due to the accumulation in the solution of inorganic anions (such as chloride, sulfates, and nitrates).

### 3.2. Characterization Results

**Figure 2:**
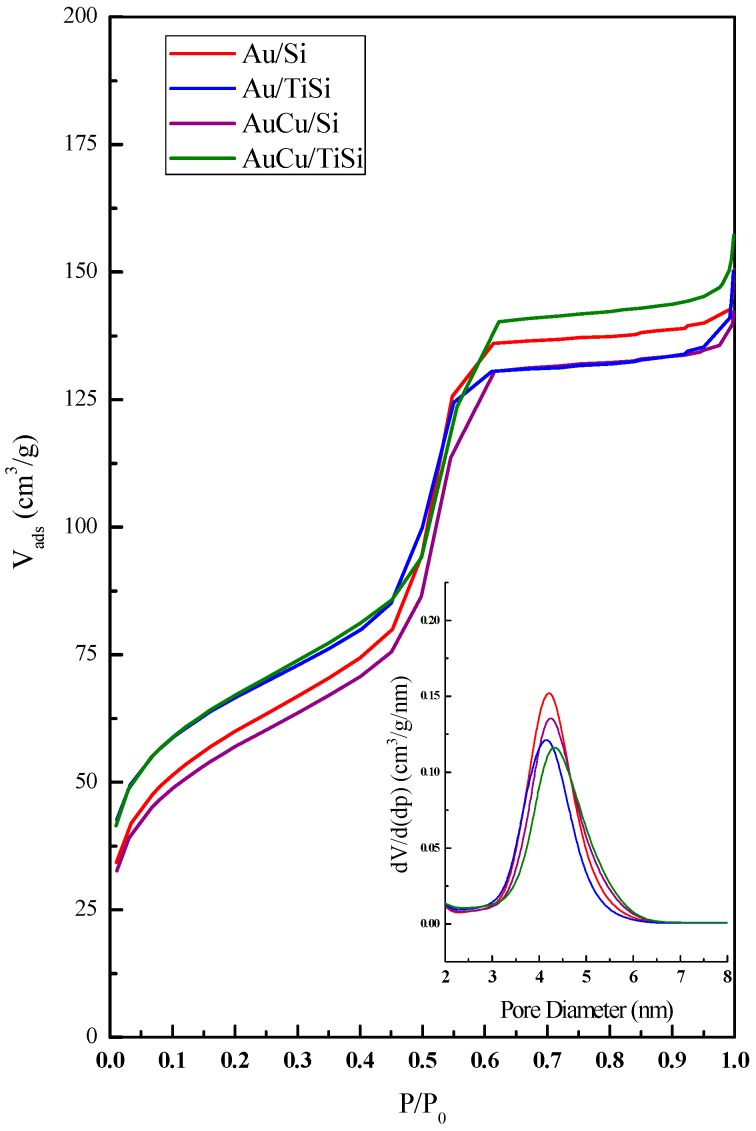
N_2_ isotherms and pore size distribution (inset) for the silica and titania-silica-based photocatalysts.

So as to evaluate the porous nature of the samples, N_2_ physisorption was carried out, and the obtained results are summarized in Table 1. The nitrogen adsorption-desorption isotherms and pore size distributions of the four samples are represented in Figure 2. They show type IV isotherm according to IUPAC classification, with a type H1 hysteresis loop distinctive of mesoporous materials like SBA-15, which is due to capillary condensation taking place in the mesopores during desorption. This fact confirms that the characteristic mesoporous structure has been maintained after the incorporation of Ti, but also after the deposition of Au and AuCu nanoparticles. In Table 1, a decrease in BET surface area can be noticed from SBA to Ti-SBA (654 m^2^·g^−1^ and 393 m^2^·g^−1^, respectively), due to the incorporation of titania that tends to block some of the pore channels of the SBA but maintaining most of them in an accessible state (inset of Figure 2). Similarly, the cumulative pore volume for SBA, 0.50 cm^3^·g^−1^, suggests a relatively large internal pore surface that diminishes by Ti incorporation with a value of 0.32 cm^3^·g^−1^. By comparing the mean pore diameter for both supports, 5.0 nm for SBA-15 and 4.8 nm for SBA-15-containing Ti, it can be stated that the wall thickness of SBA-15 remained unaltered. A significant decrease in specific surface area is observed when the metallic phases are deposited on the support, due to Au and Cu nanoparticles being incorporated inside the ordered pore channels of the mesoporous structure, thus causing a partial blockage. From these experimental observations, it could be inferred that the presence of ordered channels of the silica-based supports (Si and TiSi) leads to an effective control of the growth of the majority of Au and AuCu nanoparticles, keeping their size within the silica pore diameters (i.e., lower than 5 nm).

The diffusive reflectance UV-vis (DRUV-vis) spectra were collected in the range from 300 to 800 nm (not shown) to determine the band gap, Eg, of the photocatalysts. By comparing the Eg values of titania-containing samples (Table 1), it can be noticed that the Eg of TiO_2_ onto SBA-15, TiSi sample, is 3.13 eV, comparable to that of a bare titania. As expected, the band gap lowered significantly when Au and AuCu NPs were also present: 2.70 eV for Au/TiSi sample and 2.43 eV for AuCu/TiSi.

**Figure 3:**
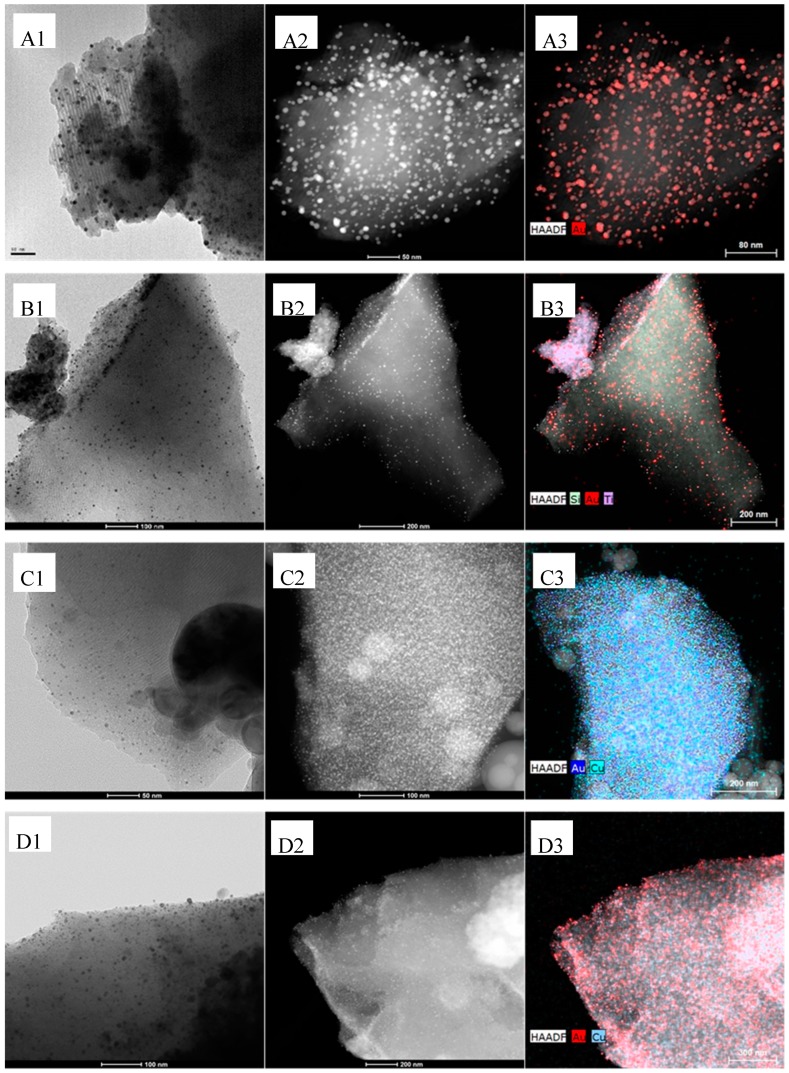
HR-TEM results corresponding to (**A**) Au/Si, (**B**) Au/TiSi, (**C**) AuCu/Si, and (**D**) AuCu/TiSi.

**Figure 4:**
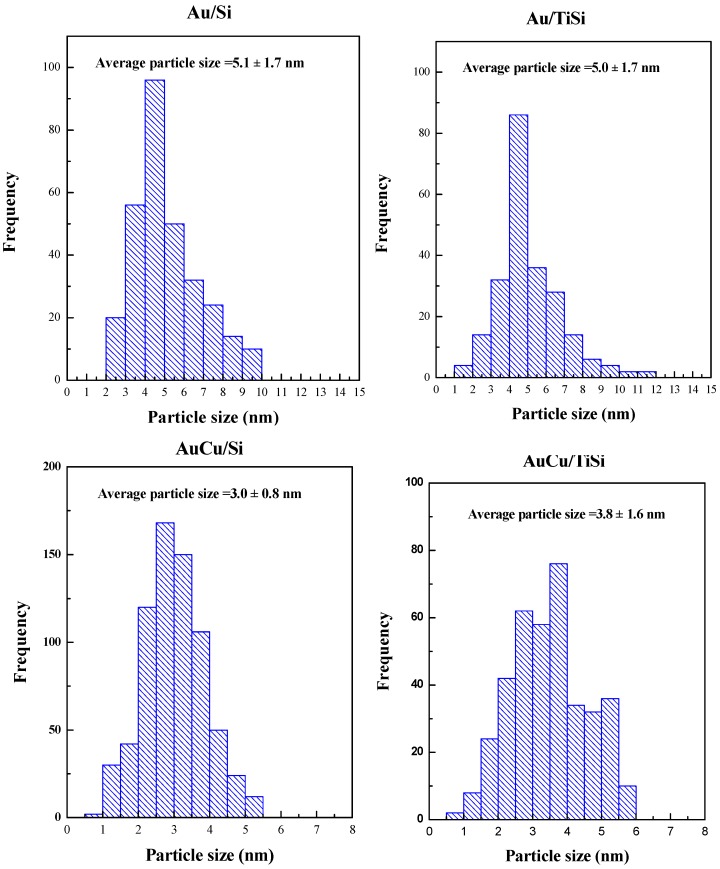
Particle size distribution obtained by TEM measurements.

In order to visually evaluate the influence of Ti and Cu on the Au particle size distribution, HR-TEM microscopy was carried out, of which images are collected in Figure 3. In agreement with XRD examinations (see below), the metal particles are found to be uniformly dispersed on the surface of the silica supports. TEM images were used to estimate the gold crystallites size distribution, as well as to investigate the influence of the second metal on the size of gold particles in bimetallic materials. In all samples, the maintenance of SBA-15 mesoporous structure, despite Ti and metal incorporation, can be clearly observed in Figure 3A1,B1,C1,D1, as inferred by N_2_ isotherms. Also, it can be easily noticed that both titania and metallic nanoparticles are incorporated and dispersed on the internal surface of SBA-15, which explains the decrease in BET surface values in Table 1. It should be noted that, although when Ti is present in the samples some agglomerates of titania are formed, as seen in Figure 3B2,D2, the metallic phase is in close contact both with the support containing Ti in its pore channels, and with titania agglomerates. Likewise, a high dispersion of the Au and AuCu alloy nanoparticles can be appreciated, especially in bimetallic samples (AuCu/Si and AuCu/TiSi), suggesting that the incorporation of Cu improves the dispersion of the metallic phase on the support. Mapping analysis has been carried out to better understand the location of each component. In the monometallic sample containing Ti, the achieved Au dispersion is lower because of the titania agglomerates, but image B3 shows a proper dispersion of Au nanoparticles and Ti on the support. Mapping results for bimetallic samples (Figure 3C3,D3) suggest, indeed, a close interaction between Au and Cu. Moreover, particle size distribution of all samples is depicted in Figure 4, where it can be observed that smaller particle sizes have been achieved in bimetallic samples: 5 nm for Au-based catalysts, and an average of 3.4 nm in AuCu-based catalysts. Thus, the addition of copper seems to improve the dispersion with the formation of smaller nanoparticles, which other authors have reported to be a critical parameter in photocatalysis [18,49,50].

**Figure 5:**
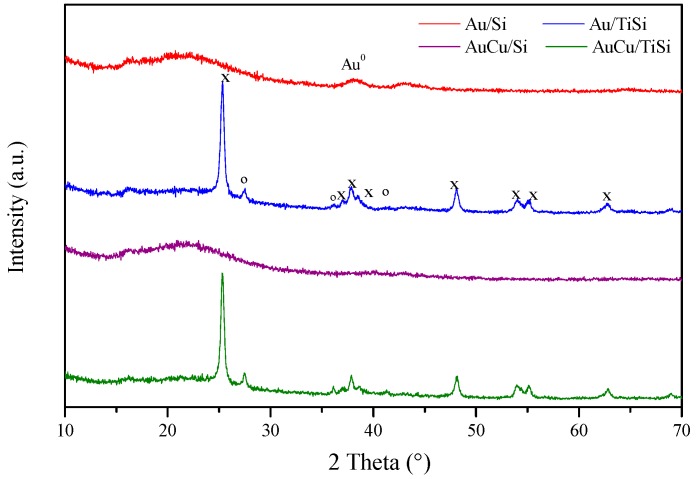
Wide angle X-ray diffractograms of the studied photocatalysts (x: anatase; °: rutile).

X-ray diffractograms at low angle (not shown) have been carried out in order to assess the maintenance of mesoporous structure after metal incorporation. From these diffractograms, d_100_ interplanar spacing was calculated, and the reflection values collected in Table 1. It is observed how after metal incorporation, d_100_ values decrease, which is indicative of metallic nanoparticles inside the channels of the SBA-15 material. When Ti is present, these values are higher, which is consistent with the location of nanoparticles in both SBA and TiO_2_ agglomerates, and therefore, a lesser proportion of Au is present in SBA mesostructure. Figure 5 displays high angle X-ray diffractograms of the prepared samples. A broad signal between 20° and 30° of 2θ characteristic of amorphous silica is observed, particularly visible in the Au/SBA sample. Regarding the Ti-containing samples, several diffraction peaks corresponding to different polymorphs can be discerned. Most of them are ascribed to anatase phase (PDF N°: 01-089-4921) at 2θ (°) = 25.3, 37.0, 37.8, 38.6, 40.1, 53.9, 55.1, and 62.8. Together with these, diffraction peaks at 2θ (°) = 27.4, 36.0, and 41.3 can be observed, related to rutile phase (PDF N°: 01-072-1148). However, no major changes were observed from these diffractograms.

**Figure 6:**
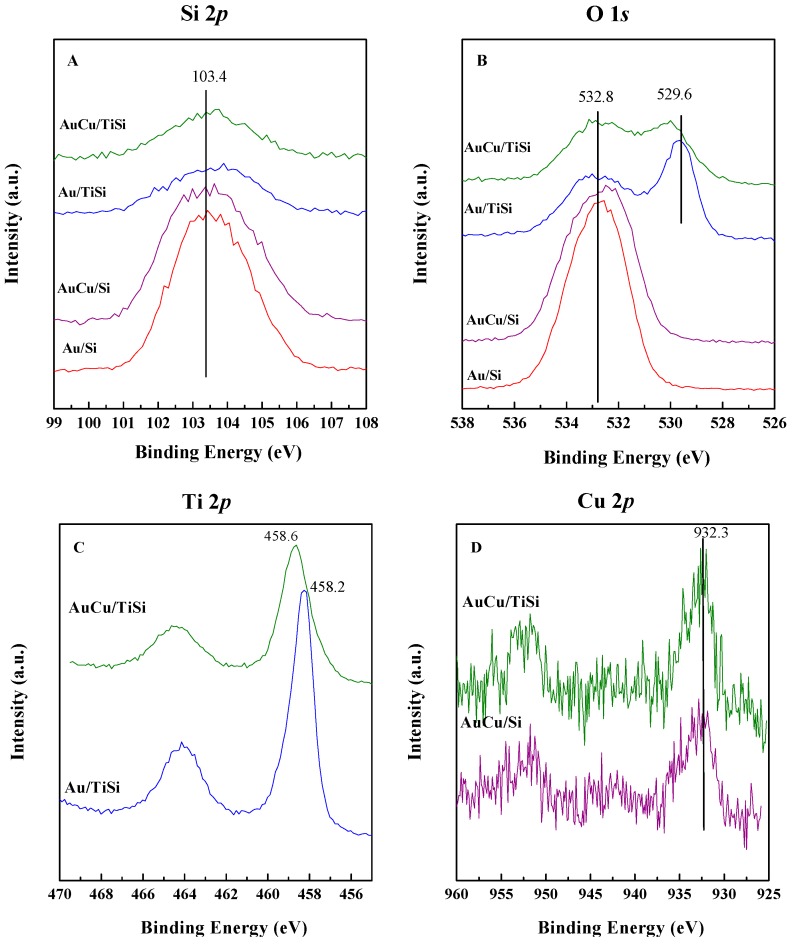
Si 2*p* (**A**), O 1*s* (**B**), Ti 2*p* (**C**), and Cu 2*p* (**D**) core level spectra of the samples.

**Figure 7:**
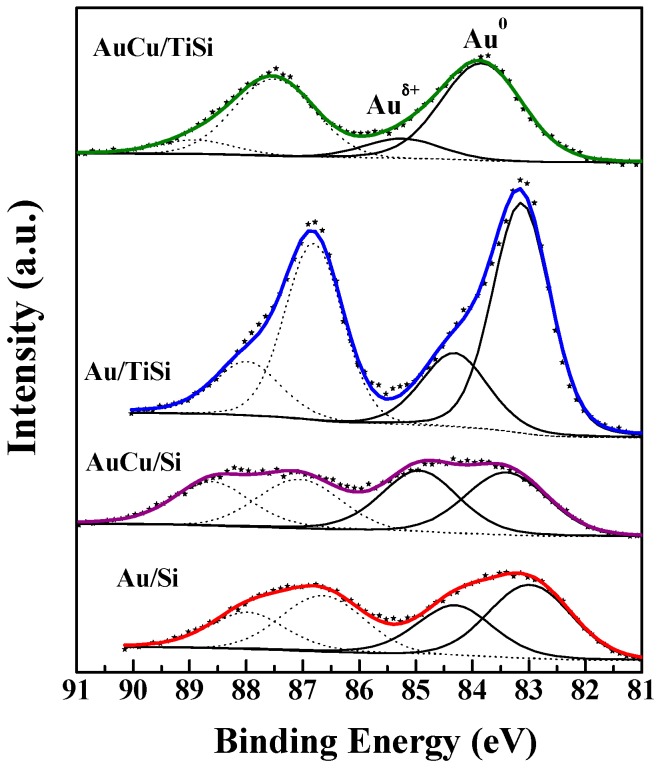
Au 4*f* XPS spectra of mono and bimetallic samples.

Information about the surface composition, oxidation states of gold and copper on the surface, as well as possible interactions between the metals, were obtained from XPS analysis, and the most relevant results are represented in Figure 6 and Figure 7, and Table 2 and Table 3. 

Si 2*p* signal is centered, in all cases, at 103.4 eV, and is attributed to SiO_2_ species [51]. The information about the state of oxygen was obtained from the O 1*s* region. The spectra of SBA samples consisted of one component at ca. 532.8 eV and, according to literature, is assigned to the oxygen in the SBA matrix [52]. Samples containing titania also possess a second contribution at ca. 529.7 eV that corresponds to TiO_2_ units [53]. Worth noting is the observed shift to higher values of this latter contribution in the AuCu/TiSi, compared to Au/TiSi counterpart, which suggests a stronger interaction between the support and the bimetallic nanoparticles in the AuCu/TiSi sample. This fact is also evident from Ti 2*p* signal, which is also shifted to higher values in the case of bimetallic sample. On the other hand, Cu 2*p* spectra, measured at low exposition time in order to avoid copper reduction by X-ray beam, are quite noisy, but the main Cu 2*p*_3/2_ peak at 932.3 eV can be distinguished, due to both Cu^0^ and Cu^1+^ species, and a shoulder typical of Cu^2+^ [54].

The deconvolution of Au 4*f* XPS spectra for the prepared photocatalysts suggests that Au species exist in two different states in all cases. Thus, two doublets corresponding to Au 4*f*_7/2_ (solid lines) and Au 4*f*_5/2_ (dotted lines) spin-orbit components are present in all the samples, and the bands possesses a Full Width at Half Maximum (FWHM) of 1.6–1.7 eV. As observed in Figure 7 and Table 2, both the position and contribution of each Au 4*f*_7/2_ component strongly depend on the catalyst formulation. In this regard, in the case of Au/Si sample, the first doublet shows the Au 4*f*_7/2_ component at 83.0 eV, and the second one at 84.3 eV. The first one implies the presence of metallic Au [55], and the second to oxidized surface species Au^δ+^ [56] that some authors have reported to be due to Au^3+^ species [57]. Au^0^ contribution for this sample corresponds to 58.8% of Au present. In the case of AuCu/Si sample, the observed shift could suggest the formation of an alloy, and the contribution of each species is hardly the same. Ti-SBA-based samples present Au signals shifted ca. 0.5 eV at higher BE compared to their SBA counterparts. A greater metal-support interaction is expected in these cases. Moreover, the formation of Au^0^ is favored in the presence of titania, and in a special way for AuCu/TiSi compound. Again, a shift in the signal after Cu incorporation was observed. The proportion of these components is given in parentheses in Table 2. It is noteworthy that both catalysts with titanium show the lowest proportion of oxidized Au.

By observing the spectra, several facts are noticeable: The Au signal is broader in SBA samples, Cu incorporation provokes a shift to higher BE values, and Ti presence favors the formation of Au^0^ species. Moreover, Ti incorporation shifts, at higher BE, the Au 4*f* signals, and does not alter the Cu 2*p* signals, and the incorporation of copper shifts Au 4*f*, Ti 2*p*, and O 1*s*, associated with titania, to higher binding energy values. 

In the case of SBA samples, the shift in the Au signal after copper incorporation could be thought as an electron transfer from Au to Cu, but considering that the work function of pure copper (4.65 eV) is lower than that of gold (5.10 eV) [58], the transfer direction has been explained as a function of the chemical state of surface elements. In this regard, the presence of some oxidized copper species on nanoparticle surface can modify the electron transfer direction between gold and copper [58]. In fact, Cu 2*p* spectra confirm this fact, and some oxidized copper species seem to be present. 

Nonetheless, after Ti incorporation, the copper signal is hardly modified, while that of Au is affected, shifting to higher values and also increasing the proportion of Au^0^, pointing to the preferential interaction of Ti with Au, rather than with Cu, caused by the electron transfer from the semiconductor to gold nanoparticles, accompanied by negative charging of Au [58]. These data suggest the preferential location of Cu on the external surface of Au nanoparticles mainly when titania is present, as noticed from the change in surface atomic composition in Table 3. The Au and Au + Cu atomic surface exposure increases in the presence of Ti, but Au surface exposure is lower after Cu incorporation, mainly in the case of Ti-SBA sample. This fact was previously suggested by Liu et al. [59] for AuCu/SBA-15 in absence of titania: the Cu component prefers to residing on the particle surface as a copper oxide layer or patches, while Au resides on the core, reasonably taking into account that the gold particles were formed prior to the deposition of copper and a core-shell structure. This distribution is proposed, but in the case of non-hydrogen reduced samples. In our case, the bimetallic samples were reduced, and the formation of AuCu alloy cannot be discarded, and the decrease of Au surface exposure was evident in the presence of Ti. Therefore, it can be thought that the preferential location of Cu on the outer surface of Au occurs in the AuCu/TiSi sample, being the most active one.

## 4. Discussion

In the present paper, we describe the photocatalytic response in methylene blue degradation of Au and AuCu catalysts supported on pure mesoporous silica and doped with titania. The general trend observed is that the presence of both titania and AuCu is of upmost importance to achieving dye decomposition within a short reaction time. Characterization results have evidenced the preferential location of metallic nanoparticles in the inner of mesoporous channels and on titania agglomerates present outside the mesoporous SBA structure. Regardless of the support, AuCu nanoparticles were lower in size than their corresponding monometallic counterparts, also observed for AuCu bimetallic systems in literature [60]. Instead, the incorporation of titanium hardly modified the mean particle size. The interaction among the different components in the catalyst formulation, as evident from XPS, suggested the formation of AuCu alloy from the shift of Au 4*f* signal to higher binding energy values, and the important role of titania in these systems was evident from the greater proportion of Au^0^, mainly in the AuCu/TiSi sample. However, the synergistic effect of AuCu is more important than the presence of Au^0^, since AuCu/Si sample is more active in the MB photodegradation than Au/TiSi, with much higher contribution of Au^0^.

The fact that TiSi samples are more active than Si ones can be explained considering that silica adsorbs the cationic dye from solution through electrostatic interaction of the surface Si–OH groups, while the titania nanocrystals provide the photoactive sites producing the OH∙ radicals required for the MB degradation, as reported by Houas et al. [41]. In a previous paper, it has been observed that for TiO_2_–SiO_2_ composites, the activity was explained considering that at a loading of titanium as high as 30 wt %, TiO_2_ nanoparticles are mainly located inside the pores, and a fraction is decorating the surface of mesoporous silica; there is, therefore, a close interaction between the sites of adsorption of MB and the sites for OH∙ production inside the mesopores [24]. A cooperative effect among TiO_2_ and SiO_2_ is required for the degradation of cationic dyes [31,61]. In this sense, the degradation of the dye was promoted by the photoactive TiO_2_ moieties, while the complete mineralization was possible, due to the confinement of intermediate products inside the mesopores. The incorporation of metal nanoparticles to TiO_2_ in MB photodegradation has also been reported in literature, mainly Au, Ag, and Cu, due to their stability in aqueous media, strong sensitizing power, and higher thermal and optical response upon irradiation with visible light, because of the surface plasmon resonance (SPR) phenomenon [62], improving the catalytic response of TiO_2_ itself. Au NPs on TiO_2_–SiO_2_ composites have also been evaluated under visible light irradiation [63]. To the best of our knowledge, no previous research has investigated methylene blue degradation under UV light using bimetallic AuCu nanocomposites. Compared to other catalytic systems, Trofimovaite et al. [64] studied the photocatalytic response in methylene blue degradation employing mesoporous TiO_2_ surface with isolated Cu(I) species, with a very low loading of Cu, and obtained a C/C_0_ ratio of ca. 0.5 after 60 min of reaction. Similarly, Chowdhury et al. [65] developed mesoporous CuO–TiO_2_ microspheres for the same reaction with C/C_0_ ca. 0.3 at the same reaction time. On the other hand, Zhang et al. [66] carried out methylene blue degradation over Au/TiO_2_ photocatalysts with C/C_0_ ~ 0.5 after 6 h of reaction. Also with Au as a metallic active phase in photocatalysis, Quiñones et al. [67] investigated methylene blue degradation under UV irradiation using Au/Pd–TiO_2_ catalysts, accomplishing a conversion of approximately 40% after 45 min of reaction. Regarding the support employed in this work, Zaccariello et al. [24] reported a remarkable efficiency for TiO_2_-mesoporous silica nanocomposites in the degradation of dyes (methylene blue and methyl orange) and drugs (paracetamol). Likewise, concerning the metallic phase used in this paper, Wen et al. [68] have studied the degradation of 2-nytrophenol over AuCu ternary composite (AuCu–P25–rGO) obtaining a C/C_0_ ratio ~0.2 at 60 min of reaction.

The set of photocatalysts examined in this work have shown outstanding results for methylene blue degradation at room temperature and ambient pressure when UV light driven (thus confirming photoresponse thereof). Especially, the sample AuCu/TiSi with a ratio *C*/*C*_0_ = 0.068 at only 60 min of reaction, so a synergistic effect between Au, Cu, and Ti could be ensured. Very small Au and AuCu bimetallic nanoparticles can act as electron relays, able to improve the MB photo-oxidation. When the NPs are in contact with TiO_2_, a Schottky barrier is formed between them, playing a paramount role in enhancing the photoactivity of the reaction [69]. As also found by other authors [23], AuCu nanoparticles deposited and incorporated inside the ordered pore channels of a SBA-15 mesoporous silica and titania-silica, proved to be effective as catalysts for the oxidation of cationic organic dyes, such as methylene blue, due to the well-known decrease of the work function of the bimetallic system with respect to the monometallic components, leading to the prevention in the recombination rate of charge carriers. Nonetheless, further thorough research should be carried out in order to understand the interaction among these species that lead to the achieved attainments.

## 5. Conclusions

Photocatalysts based on Au (1.5 wt %) and bimetallic AuCu (2.0 wt %, Au/Cu = 1), supported on SBA-15 silica with and without titania, were prepared in order to evaluate the versatility of this mesoporous support in the photocatalytic degradation of methylene blue (MB) under UV irradiation in aqueous suspension. The presence of ordered channels of the mesoporous silica and titania-silica matrixes has led to an effective control of the growth of the majority of Au and AuCu nanoparticles, keeping their size within the supports pore diameters (i.e., lower than 5 nm). Such small Au and AuCu NPs play a role in enhancing the photoactivity of the reaction. When the NPs are in contact with TiO_2_, a Schottky barrier is formed between them, thus improving charge-carrier separation and MB photo-oxidation under UV light. AuCu nanoparticles deposited and incorporated inside the ordered pore channels of SBA-15 mesoporous silica and titania-silica can be considered very effective as catalysts for the oxidation of cationic organic dyes, such as methylene blue.

## Figures and Tables

**Table 1:** Structural, textural, and optical properties of the as-prepared samples.

Sample	S_BET_ (m^2^·g^−1^)	V_p_ (cm^3^·g^−1^)	d_p_ (nm)	d_100_ (nm)	Eg (eV)
Si	654	0.50	5.0	8.74	-
TiSi	393	0.32	4.8	8.57	3.13
Au/Si	213	0.21	4.2	8.10	-
Au/TiSi	236	0.18	4.1	8.17	2.70
AuCu/Si	200	0.20	4.3	8.10	-
AuCu/TiSi	237	0.19	4.3	8.25	2.43

**Table 2:** Au 4*f_7_*_/2_ binding energy (BE) values.

	Au^0^	Au^δ+^
**Au/Si**	83.0 (58.8)	84.3 (41.2)
**AuCu/Si**	83.4 (50.8)	84.9 (49.2)
**Au/TiSi**	83.1 (72.0)	84.3 (28.0)
**AuCu/TiSi**	83.8 (84.0)	85.2 (16.0)

**Table 3:** Atomic surface composition of the samples.

Catalyst	O 1*s*	Si 2*p*	Ti 2*p*	Au 4*f*	Cu 2*p*	Au/Si	Au/(Si + Ti)	(Au + Cu)/(Si + Ti)
**Au/Si**	65.3	28.4	0.0	0.5	0.0	0.019	0.019	0.019
**AuCu/Si**	63.2	27.6	0.0	0.5	0.8	0.018	0.018	0.047
**Au/TiSi**	60.8	12.7	12.1	1.3	0.0	0.099	0.051	0.051
**AuCu/TiSi**	51.3	10.9	8.3	0.7	1.3	0.061	0.035	0.105
